# Supplementary material for: Hand hygiene of kindergarten children—Understanding the effect of live feedback on handwashing behaviour, self-efficacy, and motivation of young children: Protocol for a multi-arm cluster randomized controlled trial
Source: PLoS One. 2023 Jan 24;18(1):e0280686. doi: 10.1371/journal.pone.0280686 (PMC9873181; doi:10.1371/journal.pone.0280686)
Supplement: S1 File — (PDF) [file pone.0280686.s002.pdf]

22.11.2021 31/2021

Turun yliopiston ihmistieteiden eettisen toimikunnan
terveystieteellisten tutkimusten jaosto

ASIA: Terveystieteellisen tutkimuksen eettinen ennakkoarviointi

Tutkimuksen nimi

Candy - Lasten käsihygieniainterventio

Tutkimuksen yhteyshenkilö

Anni Pakarinen

Tutkimuksesta vastaava henkilö

Sanna Salanterä

Käsittely ihmistieteiden eettisen toimikunnan terveystieteellisten tutkimusten jaostossa

Tutkija on pyytänyt ennakkoarviointilausuntoa tutkimussuunnitelmansa eettisyydestä ja muista tutkimukseen liittyvistä riskeistä.

Turun yliopiston ihmistieteiden eettisen toimikunnan terveystieteellisten tutkimusten jaosto on 22.11.2021 kokouksessaan käsitellyt hakijan pyynnön, tutkimussuunnitelman ja siihen liittyviä asiakirjoja.

Toimikunta on pyytänyt hakijaa täydentämään hakemustaan. Hakija on toimittanut pyydetyt täydennykset.

Lausunto

Kun otetaan huomioon tutkijan pyyntö, mainitusta tutkimuksesta asiakirjoista saatava selvitys sekä Tutkimuseettisen neuvottelukunnan ohjeet ihmiseen kohdistuvan tutkimuksen eettisistä periaatteista sekä ihmistieteiden eettisestä ennakkoarvioinnista Suomessa (2019), toimikunta antaa puoltavan lausunnon. Toimikunnan käsityksen mukaan ennakkoarvioitavana oleva suunniteltu tutkimus on eettisesti hyväksyttävä.

Tämä dokumentti on allekirjoitettu sähköisesti UTUsign-järjestelmällä / This document has been electronically signed with UTUsign
Pakarinen Anni 31_21.pdf / 141370105198101112314505912924261114141429-46dd0f17fc3133801a4fd590fec7a1942f8f1bce09abb1c126ff5fc40bdc1e58 / sivu/page 1(5)

22.11.2021 31/2021

Turun yliopiston ihmistieteiden eettisen toimikunnan
terveystieteellisten tutkimusten jaosto

Varapuheenjohtaja Jarmo Niemi

Sihteeri Kirsi Klemelä

Asian käsittelyyn ovat osallistuneet Jarmo Niemi toimikunnan varapuheenjohtajana sekä Hanna Lagström, Leo Lahti, Nora Hagelberg, Ilkka Pilpola ja Susanne Uusitalo toimikunnan jäseninä. Toimikunnan sihteerinä on toiminut Kirsi Klemelä ja henkilötietojen käsittelystä vastaavana asiantuntijajäsenenä Timo Juhola.

Lisätietoja

Toimikunnan sihteeri Kirsi Klemelä,
sähköposti kirsi.klemela@utu.fi tai puh. 050 303 0346.

Muutoksenhaku

Jos ennakkoarviointilausunnon pyytäjä ei hyväksy ihmistieteiden eettisen toimikunnan päätöstä tai lausunnon sisältämiä muutosehdotuksia, hän voi pyytää asiasta lausuntoa Tutkimuseettiseltä neuvottelukunnalta. Perusteltu lausuntopyyntö liitteineen tulee jättää kahden kuukauden kuluessa eettisen toimikunnan päätöksestä.

Tämä dokumentti on allekirjoitettu sähköisesti UTUsign-järjestelmällä / This document has been electronically signed with UTUsign
Pakarinen Anni 31_21.pdf / 1413701051981011123145059129242611141429-46dd0f17fc3133801a4fd590fec7a1942f8f1bce09abb1c126ff5fc40bdc1e58 / sivu/page 2(5)

22 November 2021 31/2021

The Ethics Committee for Human Sciences at the University of Turku,
Health Care Division

MATTER: Preliminary ethical review of health care research

Title of the research project

Candy: Improving Children Hand Hygiene

Contact person for the research

Anni Pakarinen

Principal investigator of the research project

Sanna Salanterä

Handling the matter at the Health Care Division of the Ethics Committee for Human Sciences

The researcher has requested an ethical review statement on the ethicality of the research plan and on other risks related to the research.

The Health Care Division of the Ethics Committee for Human Sciences at the University of Turku has in its meeting on 22 November 2021 processed the applicant's request, the research plan, and the related documents.

The Health Care Division has asked the researcher to supplement the application. The researcher has submitted the requested amendments.

Statement

When considering the researcher's request, information about the research obtained from the delivered documents, and the national guidelines for the ethical principles of research with human participants and ethical review in human sciences, the Ethics Committee gives assent to the research. According to the Ethics Committee, the planned research project under the preliminary ethical review can be ethically approved.

University of Turku
FI-20014 University of Turku, Finland
Telephone +358 29 450 5000

Tämä dokumentti on allekirjoitettu sähköisesti UTUsign-järjestelmällä / This document has been electronically signed with UTUsign
Pakarinen Anni 31_21.pdf / 141370105198101112314505912924261114141429-46dd0f17fc3133801a4fd590fec7a1942f8f1bce09abb1c126ff5fc40bdc1e58 / sivu/page 3(5)

22 November 2021 31/2021

The Ethics Committee for Human Sciences at the University of Turku, Health Care Division

Vice Chair Jarmo Niemi

Secretary Kirsi Klemelä

The matter has been handled by Jarmo Niemi as the vice chair of the Committee and Hanna Lagström, Leo Lahti, Nora Hagelberg, Ilkka Pilpola and Susanne Uusitalo as the members of the Committee. Kirsi Klemelä was the secretary of the Committee and Timo Juhola the expert member on processing personal data.

More information

Ethics Committee secretary Kirsi Klemelä,
email. kirsi.klemela@utu.fi or tel. +358 50 3030346

Appeals

If the applicant for the statement does not accept the decision of the Ethics Committee for Human Sciences or the proposed changes in the statement, the researcher may request a statement from the Finnish Advisory Board on Research Integrity (TENK). The request for statement with justifications and appendices has to be submitted within two months of the decision of the Ethics Committee.

Tämä dokumentti on allekirjoitettu sähköisesti UTUsign-järjestelmällä / This document has been electronically signed with UTUsign
Pakarinen Anni 31_21.pdf / 14137010519810111231450591292426111414l429-46dd0f17fc3133801a4fd590fec7a1942f8f1bce09abb1c126ff5fc40bdc1e58 / sivu/page 4(5)

Tämä dokumentti on allekirjoitettu sähköisesti Turun yliopiston UTUsign-järjestelmällä
This document has been electronically signed with the UTUsign system of the University of Turku

Päiväys / Date: 02.12.2021 16:45:16

Jarmo Niemi
lehtori
Turun yliopisto
Kaksiosainen henkilötunnistus (Sähköposti- ja puhelintunnistus)
Two-factor person identification (E-mail and SMS identification)

Päiväys / Date: 02.12.2021 16:09:08

Kirsi Klemelä
kehittämisasiantuntija
Turun yliopisto
Kaksiosainen henkilötunnistus (Sähköposti- ja puhelintunnistus)
Two-factor person identification (E-mail and SMS identification)
