# Supplementary material for: Hand hygiene of kindergarten children—Understanding the effect of live feedback on handwashing behaviour, self-efficacy, and motivation of young children: Protocol for a multi-arm cluster randomized controlled trial
Source: PLoS One. 2023 Jan 24;18(1):e0280686. doi: 10.1371/journal.pone.0280686 (PMC9873181; doi:10.1371/journal.pone.0280686)
Supplement: S2 File — (PDF) [file pone.0280686.s003.pdf]

Otto-Friedrich-Universität Bamberg

Otto-Friedrich-Universität Bamberg • Ethikrat • 96045 Bamberg

Prof. Dr. Thorsten Staake
Lehrstuhl für Wirtschaftsinformatik,
insbesondere Energieeffiziente Systeme
An der Weberei 5
D-96047 Bamberg

Ethikrat
Otto-Friedrich-Universität
Bamberg

Prof. Dr. Thomas Weißer (Laubach)

Tel. +49 (0) 951 / 863 1733
Fax +49 (0) 951 / 863 4734
ethikrat@uni-bamberg.de

Bamberg, den 17.01.2022

Ethics Statement

Dossier number 2021-12/58

Dear colleagues,

The study

Candy - Children Hand Hygiene, Full scale study

was reviewed by the Ethics Committee of the University of Bamberg, Germany, and an approval granted.

With best regards

Prof. Dr. Thomas Weißer (Laubach)

Chairman of the Ethics Committee

BESUCHSADRESSE
Otto-Friedrich-Universität Bamberg
An der Universität 2
Raum U2/02.11
96047 Bamberg

BRIEFADRESSE
Otto-Friedrich-Universität Bamberg
96045 Bamberg

Otto-Friedrich-Universität Bamberg

Otto-Friedrich-Universität Bamberg • Ethikrat • 96045 Bamberg

Prof. Dr. Thorsten Staake
Lehrstuhl für Wirtschaftsinformatik,
insbesondere Energieeffiziente Systeme
An der Weberei 5
D-96047 Bamberg

Ethikrat
Otto-Friedrich-Universität
Bamberg

Prof. Dr. Thomas Weißer (Laubach)

Tel. +49 (0) 951 / 863 1733
Fax +49 (0) 951 / 863 4734
ethikrat@uni-bamberg.de

Bamberg, den 17.01.2022

Ethics Statement

Dossier number 2021-12/58

Dear colleagues,

The study

Candy - Children Hand Hygiene, Full scale study

was reviewed by the Ethics Committee of the University of Bamberg, Germany, and an approval granted.

With best regards

Prof. Dr. Thomas Weißer (Laubach)

Chairman of the Ethics Committee

BESUCHSADRESSE
Otto-Friedrich-Universität Bamberg
An der Universität 2
Raum U2/02.11
96047 Bamberg

BRIEFADRESSE
Otto-Friedrich-Universität Bamberg
96045 Bamberg
