# Supplementary material for: Hand hygiene of kindergarten children—Understanding the effect of live feedback on handwashing behaviour, self-efficacy, and motivation of young children: Protocol for a multi-arm cluster randomized controlled trial
Source: PLoS One. 2023 Jan 24;18(1):e0280686. doi: 10.1371/journal.pone.0280686 (PMC9873181; doi:10.1371/journal.pone.0280686)
Supplement: S3 File — (PDF) [file pone.0280686.s004.pdf]

Candy: Improving Children Hand Hygiene

Content

1. Brief description of the study ... 3
2. Motivation & related work ... 3
3. Objectives ... 4
4. Significance of the research ... 5
5. Approach and methodology ... 6
6. Ethical considerations ... 9
7. Workplan / timeline ... 9

8. Research team 10

9. List of references 11

1. Brief description of the study

Candy project targets children's hand hygiene in a novel way by developing, evaluating, implementing smart IoT-based, and gamified intervention. The project involves four phases which are baseline, intervention, post-intervention, and follow-up phases. The aim of this full-scale study (randomized controlled trial) is to evaluate the effectiveness of gamified digital intervention among preschool children (3-6 years old) in achieving better hand hygiene. The digital intervention includes an easy-to-implement, real-time digital feedback provided during hand hygiene process in kindergartens ("live feedback"). The findings of this study will inform the development of faucets that can serve as "digital personal trainers", costing only little more than conventional devices and are suitable for installations in kindergartens, hospitals, elderly care facilities, and public buildings.

Keywords: Hand washing; real-time feedback; behavioral control; kindergarten; education for children; habit formation

2. Motivation & related work

Good hand hygiene is a crucial factor in preventing the spread of respiratory and gastrointestinal diseases (Aiello et al., 2008). Poor hand hygiene has high social costs in the private sector, with a high number of sick days and a significant burden on individuals, employers and the welfare system being attributed to infections that could have been prevented (WHO, 2011). Consequently, considerable efforts should be made to improve hand hygiene in general especially during the pandemics. In day care centers, the risk of infections is immense (Brady, 2005) as children play closer to the ground and are often exposed through hand to mouth activities (WHO, 2021).

Children are actors in their own lives already at young age and are fully capable of learning and internalizing health information. Kindergartens are viable settings for targeting young children for educational interventions. (See e.g., Ramseier et al., 2007; Rosen et al., 2006.) Educational interventions targeting children should be offered in a child-centered and age-appropriate way to support understanding and motivation to learn (Prensky 2001).

Gamification offers one viable method for education among children. Gamification, with carefully designed game elements, affects learning via mediation, e.g., through targeting behavior (Nand et al., 2019). Motivation, positive reinforcement, and observational modelling are playing important roles in learning and behavior changes and children with higher sense of self-efficacy (*belief in one's capabilities*) tend to have better motivation (Bandura 2004). Thus, social cognitive theory provided the theory base for our study to support the success of the intervention among children.

Nevertheless, research on kindergarten hand hygiene and hygiene in general is scarce and often not well executed (Wang et al., 2017; Willmott et al., 2016), unlike hand hygiene in hospitals, where numerous studies exist (see e.g. WHO, 2004). The few existing studies researching hygiene in an educational setting (e.g., Liu et al., 2019) are limited in a way that (i) the presented hygiene measures are hardly implementable on a day-to-day basis and (ii) it is questionable if the presented behavioral interventions have a long-term impact.

Ofori et al. (2020) present three technical solutions (computer games, educational videos and video camera observation) that have been used to promote hygiene in educational settings of all age groups. While all three solutions show positive short-term effects on hand-washing behavior, none of the solutions investigate long-term effects. Furthermore, existing studies are not focused on behavioral effects. Azor-Martinez et al. (2018), for example, outline the positive effect of hand hygiene in regards to the reduction of sick days but omit to observe which part of the multicomponent intervention really triggers behavior changes.

To overcome the mentioned shortcomings, we intend to test the effectiveness of a digital intervention that is provided automatically by faucets used for hand washing in kindergartens and which aims to improve hygiene through presenting empowering feedback to children. Thus, we use gamification to motivate, educate, increase handwashing self-efficacy, and affect behavior of children (Baranowski et al., 2016; Hamari et al., 2014; Morschheuser et al., 2018; Prensky 2001). The digital intervention is designed to allow for large-scale implementations (unlike video camera observations that are sometimes used in hospitals' operation theaters), to give immediate feedback at the point of action (unlike educational videos) and that can be maintained indefinitely (unlike personal training). Since hand washing can be seen as one of the most effective measure to prevent infection transmission (CDC, 2002), we focus our efforts on the hand-washing behavior. Furthermore, since parents and their socio-economic status show considerable effect on health-related behavior of children (Branden et al., 2012; Song et al., 2013), we implement our measures in kindergartens, to reach children with a large variety of backgrounds.

More specifically, we will vary the availability of real-time information on hand washing during the behavior ("live feedback") and evaluate the behavioral response of the children. We argue that real-time feedback during an action - an approach that is becoming increasingly possible with the advent of IoT technology - is effective in improving hand hygiene on a large scale. Moreover, the collected behavioral data allow a better understanding of the response of children to such interventions and can help to understand the barriers that exist to achieve better behavior also beyond the specific application and context.

3. Objectives

The aim of this full-scale study (randomized controlled trial) is to evaluate the effectiveness of gamified digital intervention among kindergarten children (3-6 years old) in achieving better hand hygiene. The digital intervention includes an easy-to-implement, digital feedback provided during hand hygiene in kindergartens ("live feedback").

The research design will allow us to pursue the following objectives:

1. The first objective is to determine the effectiveness of live feedback on hand washing behavior of children and the effect's short and long-term stability. The results provide a first indication of the practical value of the intervention in the context under study.

2. Second, we intend to determine the duration it takes for the effect to unfold. The findings will reveal if a learning phase is required or if live feedback is effective from the onset.
3. The third objective is to provide a basis for future work that tests if the effects replicate also under different settings.

4. Significance of the research

The study brings a randomized controlled trial into a productive field environment and collects real-time data for many installations in kindergartens and over a long duration of (3 months). It thus combines the strength of the networked, sensor-equipped "everyday-products" from the emerging Internet of Things (IoT) with the methodological strengths of behavioral research. Thereby, the project demonstrates how IoT can bring behavioral research from lab to field while maintaining person-specific interventions but with increased reach/scalability of the measure and with the opportunity to collect high-quality data. It also eliminates observer biases (see e.g., (Srigley et al., 2014) as the IoT-faucets collect the data anonymously. The project also combines an investigation of a rather practical problem (hand hygiene) with more basic research on the mechanism of live feedback interventions.

Despite the extensive work on feedback in general, research on live feedback is sparse, especially if it addresses routine tasks over longer periods and takes place as field experiment. The very large effect size of live feedback which has been observed earlier (e.g., (Tiefenbeck et al., 2016) calls for studies in different settings. The findings of this study allow getting insights on the effect of live-feedback on hand-washing behavior of children. Thus, it enables to derive statements about live-feedback in general and hand-washing behavior of children in specific. In addition, even gamification has shown to be an effective approach in educational interventions among children (Nand et al., 2019), there is to a lesser extent of studies that have been explored the phenomena among very young children. Thus, the findings of this study will bring more insight of the value of gamification among this age group.

The findings will also address major societal challenges regarding and beyond the current pandemic as good hand hygiene prevents the spread of many other infectious diseases which constitute a high burden for individuals and the economy. For the society in general, insight on the mechanics of live feedback is relevant simply because related interventions will become possible for a plethora of tasks related to health, energy consumption, risky behavior, and education due to the development of sensor technologies and the emerging Internet of Things. The potential power of live feedback calls for a better understanding of its mechanisms and effects.

In the future, the described study could be extended to investigate a larger number of interventions and their effect on self-efficacy and attitudes. The experimental set-up would be changed in a way, that an additional group of children would have the ability to choose the motivator displayed on the screen. In addition to that, another study could focus on habit formation, examining the duration of habit formation and the persistence of habit which is only seldom investigated in large field trials.

5. Approach and methodology

Study design

We conduct an effectiveness study (randomized controlled trial) with three groups; the control group, instruction group, and reward group in four different phases (baseline, intervention, post-intervention, and follow-up). While the control group does not get any feedback from the system (no screen activity over the course of the study), the instruction group will receive instructions from the screen display but no reward during the intervention phase (Figure 1), and the reward group will receive instructions and reward shown on a screen display right next to the sink during the intervention phase (Figure 2). The reward will be a random choice of different motivating images shown if the hand are washed correctly (water and soap usage). The success of these motivators will be tested with children of the respective age beforehand. All children will receive a "how-to" teaching session (video-based training) on hand washing for the children to learn the same knowledge about hand-washing.

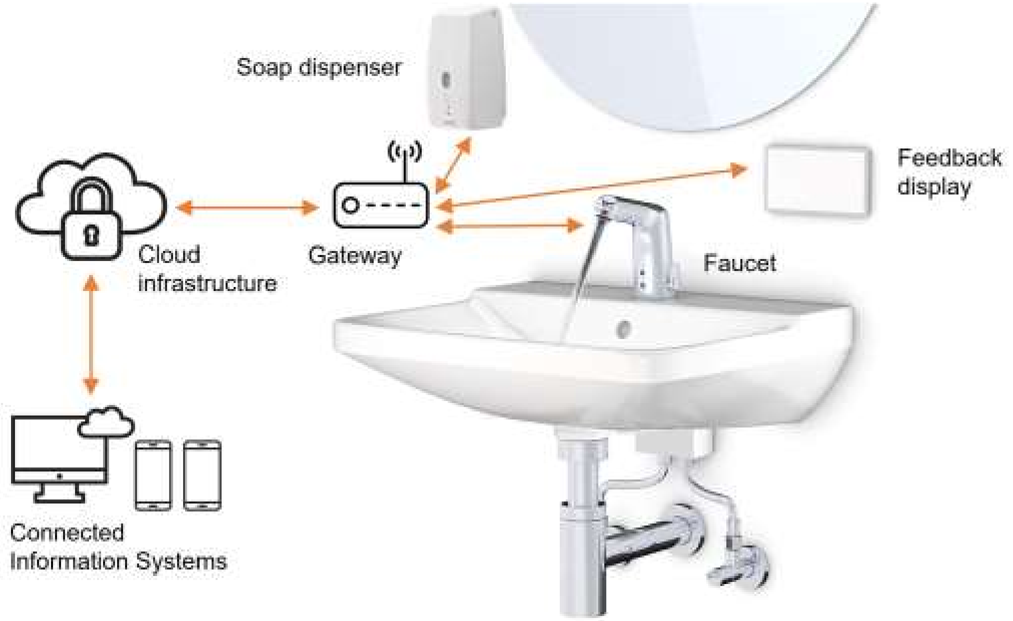

Figure 1: Mockup of sink equipped with a feedback display

Intervention procedures

The study will be conducted in four phases: the baseline phase, the intervention phase, the post-intervention phase, and follow-up phase (see Figure 2).

- **Baseline Phase:** The experiment is preceded by a baseline phase of **2 -3 weeks** when the system is installed but does not provide any feedback or other information (no screen activity). To ensure that all kids have the same knowledge about handwashing and to enable them to wash their hands correctly, a "how-to" teaching session (video-based training) will be held in each kindergarten after half of the baseline phase has passed.

- **Intervention Phase:** The treatment phase will last about **4 weeks**. While the control group does not get any feedback (no screen activity), the instruction group gets instruction (how to do handwashing) with no reward, and reward group receive instruction and if hands are washed correctly (water and soap usage) a reward (animal animation) will be shown on the display for each handwashing activity.
- **Post-intervention Phase**: The post-treatment phase is followed by **3 weeks** where only data from the system is still collected but the display is deactivated for the instruction and reward groups, wherein no screen activity in all groups. The system will be deinstalled at the end of the post-intervention phase.
- **Follow-up phase:** The follow-up phase is followed by **6 months** after the post-intervention phase wherein there is no data collected from the system in all groups. In this phase, the number of sick leave days of the children and staff in the kindergarten will be collected.

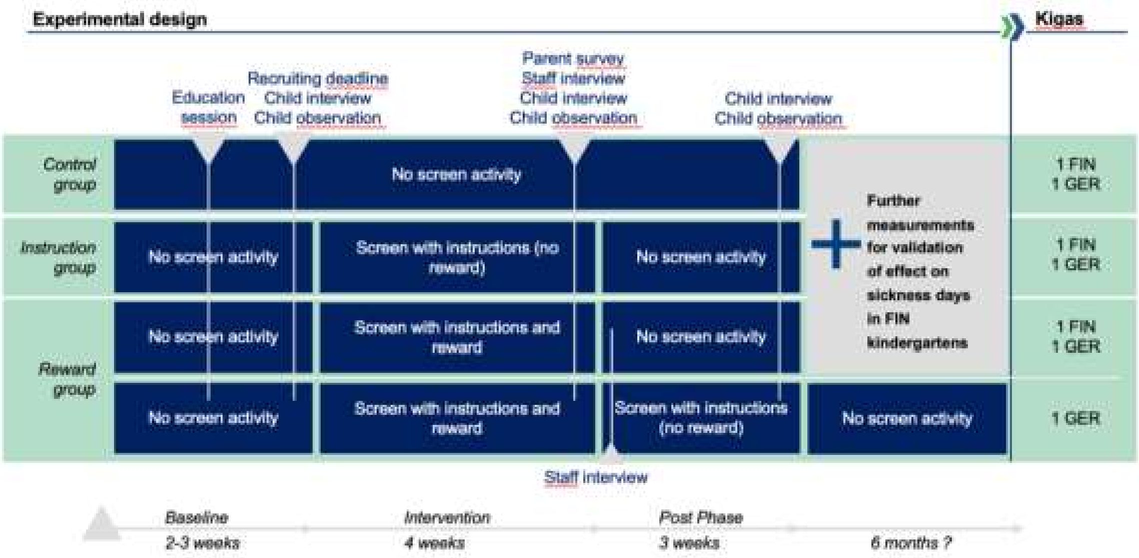

Figure 2: Overview of intervention procedure

Sample

About three to five kindergartens in Finland and in Germany will be chosen to participate. The exact number of participating kindergartens depends on the size / number of children per kindergarten, which is expected to be around 50 to 100 children. In total, around 10.000 hand washing procedures will be collected. For the randomization, we will use cluster randomized design, where one kindergarten serves as one cluster. Kindergartens are randomly assigned (by computer software program) to any of the groups. The sample size will be calculated based on the pilot study using power calculation.

Data collection

The data will be collected in four phases: the baseline, the intervention, the post-intervention phase, and follow-up. Data will be analyzed using qualitative methods and statistical methods.

Measured variables:

For each water extraction, the following variables will be measured:

- Start time of extraction, water volume, water temperature, end time of extraction; from this data, the following data will be derived: average flow rates, approximation of energy consumption (Baseline, Intervention, and Post-intervention phases)
- Quality of hand washing; duration of hand washing, time stamp of soap use, time stamp of paper towel use (Baseline, Intervention, and Post-intervention phases)
- Type of feedback given via a local display
- ID of the faucet#

The following data will be collected per participating child, from the parents, and the kindergarten staff:

- Per participating child: Age and gender, perceived self-efficacy, observed attitude, and handwashing confidence (Interviews and Observations 1,2, & 3, and sick leave days in follow-up phase; fig 2)
- From the parents: Children's observed handwashing behavior at home (Survey 1; fig 2)
- From the kindergarten staff: Children's observed behavior and the quality of hand washing routines at kindergarten (Interviews 1 & 2, and sick leave days in follow-up phase; fig 2)

Data collection

The variables regarding the water extraction will be measured with an existing infrastructure that has been extensively tested in other environments (e.g., hospitals). The system is installed beneath the sink, so it is invisible to users and the faucet is upgraded to a digital faucet that comes into a similar appearance than a standard contactless faucet. The display that shows the feedback interventions is a low-cost but high-contrast monochromatic e-ink display about 50 x 120 mm in size that is directly in sight when washing hands. Data is collected separately for each extraction. For comparability of the results, it is of importance that all washbasins, soap, and towel dispenser are at a height adequate for kindergarten children.

The data collection from the children will be their self-reported confidence regarding their hand washing performance which will be collected through one-on-one interview and observed attitude and handwashing performance through observation. For the parents, their perceptions on child hand hygiene behaviors will be collected with surveys. The surveys of parents and their respective child

will be matched with an identifier. The data regarding the kindergarten staff's perceptions on children's hand hygiene behaviors will be collected through interview. Pre-tests will be done for the children's interview, parent survey, and kindergarten interview contents to the target participants of a sample size of 3 to 5. A possible modification in the questionnaires and surveys will be done after analyzing the result of the pre-test.

Data protection

See comprehensive data management plan (attachment 6).

6. Ethical considerations

Included in attachment 2.

7. Workplan / timeline

CW 33-36: Preparation of Data Management Plan, detailing of Study Design and feedback intervention (incl. display), drafting of an information letter for the family and an agreement of consent

CW 36-39: Refinement of the study design and programming of the interventions

CW 37-42: Development of pre-survey for parents and pre-testing of motivators / feedback with children, interview questions, and observation plan

CW 43: Presentation in ethics committee, University of Turku

CW 44: Permission to conduct the study from the municipalities

CW 45-46: Test-run of the final intervention in University Building

CW 46-47: Pre-test of scale measurement and Recruitment and randomization of kindergartens

CW 48-51: Recruitment of participants and acquiring of informed consents

CW 2: Installation of digital faucets, Installation of the data collection modules and communication gateway

CW 3: Training of the kindergarten staff (intervention procedures)

CW 2-5: Baseline measurement, hardware maintenance, educational training/video in kindergartens

CW 6-10: Intervention phase + Development of ex-post-survey for parents and preparation of interview guideline for kindergarten staff

CW 11-13: Post-intervention phase, data from the system

CW 14: Deinstallation of measurement devices

CW 15-21: Data analysis and reporting

CW 22-26: Fine tuning, discussion of findings with external researchers, etc.

CW 40-41: Collection of sick leave days of children and staff

8. Research team

The research team consists of five researchers from the University of Turku and four researchers from the University of Bamberg.

University of Bamberg:

- Thorsten Staake is professor of Information Systems (IS) at the University of Bamberg. His research focuses on digital interventions and personal decision support systems. He has published results from methodologically similar field studies in leading journals of the IS and Energy discipline, including Management Science and Nature Energy, and results on feedback interventions have been featured as highlight in Nature.
- Joanna Graichen is a PhD student at Thorsten's chair and will be the operational lead for the presented study.
- Carlo works fulltime as PhD student at Thorsten’s chair. He has extensive experience with the infrastructure and is running a large-scale experiment about hand-washing behavior in hospitals.
- Sebastian Günther is a high-tenured PhD student at Thorsten’s chair. Sebastian has successfully run complex field studies on digital interventions before, with results published in leading IS Conferences (e.g., ICIS 2017) and forthcoming in Global Environmental Change.

University of Turku:

- Sanna Salanterä is professor of Clinical Nursing Science at the University of Turku. Sanna is co-leader of research programme Connected Health. Sanna brings to the project extensive experience in health studies and randomized controlled trials in health care environments.
- Anni Pakarinen (RN, MHSc, PhD) will be the operational lead for the presented study. She is senior researcher and development manager at Department of Nursing Science at the University of Turku. Her research focuses on health promotion and digital interventions, particularly on serious games and gamified applications. She has experience in user-centered design process, development of digital interventions, testing usability and feasibility of interventions and effectiveness studies.
- Riitta Mieronkoski (physiotherapist, MHSc) is PhD student at Salanterä’s research team Connected Health. Her expertise is in sensor data research in the area of health.

- Kirsi Terho (RN, ICN, MNS) is PhD student at Salanterä's research team Connected Health. Her research area is in infection prevention.
- Glenda Dangis (RN) is a master's student at Salanterä's research team Connected Health. Her research focuses on health and technology.
- Antti Siloaho is a master's student at Salanterä's research team Connected Health.

9. List of references

Aiello, A. E., Coulborn, R. M., Perez, V., & Larson, E. L. (2008). Effect of Hand Hygiene on Infectious Disease Risk in the Community Setting: A Meta-Analysis. *American Journal of Public Health*, *98*(8), 1372–1381. https://doi.org/10.2105/AJPH.2007.124610

Azor-Martinez, E., Yui-Hifume, R., Muñoz-Vico, F. J., Jimenez-Noguera, E., Strizzi, J. M., Martinez-Martinez, I., Garcia-Fernandez, L., Seijas-Vazquez, M. L., Torres-Alegre, P., Fernández-Campos, M. A., & Gimenez-Sanchez, F. (2018). Effectiveness of a Hand Hygiene Program at Child Care Centers: A Cluster Randomized Trial. *Pediatrics*, *142*(5). https://doi.org/10.1542/peds.2018-1245

Bandura, A. (2004). Health promotion by social cognitive means. Health education & behavior, 31(2), 143-164.

Baranowski, T., Blumberg, F., Buday, R., DeSmet, A., Fiellin, L. E., ... & Mellecker, R. Institute of Digital Media and Child Development Working Group on Games for Health, (2016). Games for health for children—Current status and needed research. Games for health journal, 5(1), 1-12.

Branden, S. V. den, Broucke, S. V. den, Leroy, R., Declerck, D., & Hoppenbrouwers, K. (2012). Effects of time and socio-economic status on the determinants of oral health-related behaviours of parents of preschool children. *European Journal of Oral Sciences*, *120*(2), 153–160. https://doi.org/10.1111/j.1600-0722.2012.00951.x

CDC. (2002). Guideline for Hand Hygiene in Health-Care Settings. *Morbidity and Mortality Weekly Report*. Centers for disease control and prevention.

Hamari, J., Koivisto, J., & Sarsa, H. (2014, January). Does gamification work?--a literature review of empirical studies on gamification. In 2014 47th Hawaii international conference on system sciences (pp. 3025-3034). Ieee.

Heider KL, Jalongo MR. (2014). Young Children and Families in the Information Agge: Applications of Technology in Early Childhood. Vol 10. Springer Netherlands. https://ebookcentral.proquest.com/lib/kutu/detail.action?docID=1968136

Liu, X., Hou, W., Zhao, Z., Cheng, J., van Beeck, E. F., Peng, X., Jones, K., Fu, X., Zhou, Y., Zhang, Z., Richardus, J. H., & Erasmus, V. (2019). A hand hygiene intervention to decrease hand, foot and mouth disease and absence due to sickness among kindergarteners in China: A cluster-randomized controlled trial. *Journal of Infection*, *78*(1), 19–26. https://doi.org/10.1016/j.jinf.2018.08.009

Morschheuser B, Hassan L, Werder K, Hamari J. How to design gamification? A method for engineering gamified software. Information and Software Technology. 2018 Mar 1;95:219-37.

Nand, K., Baghaei, N., Casey, J., Barmada, B., Mehdipour, F., & Liang, H. N. (2019). Engaging children with educational content via Gamification. Smart Learning Environments, 6(1), 6.

NAEYC and Fred Rogers Center. (2012). Technology and Interactive Media as Tools in Early Childhood Programs Serving Children from Birth through Age 8. Published online 2012. https://www.naeyc.org/sites/default/files/globally-shared/downloads/PDFs/resources/position-statements/ps_technology.pdf

Ofori, S. K., Hung, Y. W., Schwind, J. S., Muniz-Rodriguez, K., Kakou, R. J., Alade, S. E., Diallo, K., Sullivan, K. L., Cowling, B. J., & Fung, I. C. H. (2020). The use of digital technology to improve and monitor handwashing among children 12 years or younger in educational settings: A scoping review. *International Journal of Environmental Health Research*, 1–18. https://doi.org/10.1080/09603123.2020.1784398

Prensky M, Digital Game-Based Learning (McGraw Hill, New York, 20019Brady, M. T. (2005). Infectious disease in pediatric out-of-home child care. *American Journal of Infection Control*, *33*(5), 276–285. https://doi.org/10.1016/j.ajic.2004.11.007

Ramseier, C. A., Leiggener, I., Lang, N. P., Bagramian, R. A., & Inglehart, M. R. (2007). Short-term effects of hygiene education for preschool (kindergarten) children: a clinical study. Oral health & preventive dentistry, 5(1).

Rosen, L., Manor, O., Engelhard, D., Brody, D., Rosen, B., Peleg, H., ... & Zucker, D. (2006). Can a handwashing intervention make a difference? Results from a randomized controlled trial in Jerusalem pre-schools. Preventive medicine, 42(1), 27-32.

Song, I. H., Kim, S.-A., & Park, W.-S. (2013). Family factors associated with children's handwashing hygiene behavior. *Journal of Child Health Care*, *17*(2), 164–173. https://doi.org/10.1177/1367493512456106

Srigley, J. A., Furness, C. D., Baker, G. R., & Gardam, M. (2014). Quantification of the Hawthorne effect in hand hygiene compliance monitoring using an electronic monitoring system: A retrospective co-hort study. *BMJ Quality & Safety*, *23*(12), 974–980. https://doi.org/10.1136/bmjqs-2014-003080

Tiefenbeck, V., Goette, L., Degen, K., Tasic, V., Fleisch, E., Lalive, R., & Staake, T. (2016). Overcoming Salience Bias: How Real-Time Feedback Fosters Resource Conservation. *Management Science*, *64*(3), 1458–1476. https://doi.org/10.1287/mnsc.2016.2646

Wang, Z., Lapinski, M., Quilliam, E., Jaykus, L.-A., & Fraser, A. (2017). The effect of hand-hygiene interventions on infectious disease-associated absenteeism in elementary schools: A systematic literature review. *American Journal of Infection Control*, *45*(6), 682–689. https://doi.org/10.1016/j.ajic.2017.01.018

WHO. (2004). WHO News: Hand washing could save the lives of millions of children. *Bulletin of the World Health Organization*, *82*(8), 631–633.

WHO. (2011). *Report on the Burden of Endemic Health Care-Associated Infection Worldwide*. https://apps.who.int/iris/bitstream/handle/10665/80135/9789241501507_eng.pdf

WHO. (2011). Children are among the most vulnerable to environmental threats - Advocacy publications. Published 2021. https://www.who.int/heca/advocacy/publications/HECIbr2.pdf

Willmott, M., Nicholson, A., Busse, H., MacArthur, G. J., Brookes, S., & Campbell, R. (2016). Effectiveness of hand hygiene interventions in reducing illness absence among children in educational settings: A systematic review and meta-analysis. *Archives of Disease in Childhood*, *101*(1), 42–50. https://doi.org/10.1136/archdischild-2015-308875
